# First experience with Walrus balloon guide catheter in a whole-body flow model

**DOI:** 10.1007/s00234-023-03214-w

**Published:** 2023-08-29

**Authors:** Helena Guerreiro, Fabian A Flottmann, Anna A. Kyselyova, Maximilian Wagner, Caspar Brekenfeld, Bernd Eckert, Till Illies, Fritz Wodarg, Jens Fiehler, Maxim Bester

**Affiliations:** 1 Department of Diagnostic and Interventional Neuroradiology, University Medical Center Hamburg-Eppendorf, Martinistraße 52, 20251 Hamburg, Germany; 2 Department of Radiology and Neuroradiology, Asklepios Klinik Altona, Hamburg, Germany; 3 Department of Radiology and Neuroradiology, University Hospital Schleswig-Holstein, Kiel, Germany

**Keywords:** Stroke, Thrombectomy, Balloon, Catheter, Intervention

## Abstract

**Purpose:**

Flow arrest using a balloon guide catheter (BGC) in mechanical thrombectomy (MT) due to large vessel occlusion has been associated with better outcomes. Known limitations of currently commercially available BGCs are incompatibility with large bore aspiration catheters (AC) and lack of distal flexibility. Walrus presents variable stiffness and compatibility with large bore AC. The goal of this study is to describe the first experience with Walrus in a realistic stroke simulation model.

**Methods:**

A full-length modular vascular model under physiological conditions was used. 8F^+^-Walrus inner-diameter (ID) 0.087in 95 cm combined with 6F-Sofia AC ID 0.070in 131 cm and an 8F-Flowgate2 BGC ID 0.084in 95 cm with a 5F-Sofia AC ID 0.055in 125 cm were used to perform aspiration MT. User surveys, access to target and occlusion site, technique, time of delivery, anatomical change, and catheter kick-back were assessed.

**Results:**

Seven neuroradiologists with average of 10 years-experience in MT performed primary aspiration using the above-mentioned combinations in three different anatomies (*N* = 41). All operators would likely (29%) or very likely (71%) use again Walrus in combination with large bore AC and the majority (86%) found its navigability easier than with other BGCs. Time to reach final BGC position and catheter kick-back did not differ significantly among anatomies or catheter combinations (*p* > 0.05). However, Walrus was more likely to reach ICA petrous segment (*p* < 0.05) and intracranial occlusion with AC (*p* < 0.01).

**Conclusion:**

The Walrus combined with large bore AC presented significantly better distal access and navigability for primary aspiration in an in vitro stroke model.

## Introduction

The endovascular treatment of large vessel occlusion (LVO) in acute ischemic stroke (AIS) has shown irrefutable evidence as the treatment of choice for selected patients [1–4]. Different technical approaches have been suggested, yet global unanimity remains to be achieved. A direct aspiration first pass technique (ADAPT) is often considered a quick and effective first line approach [5]. A combined technique with aspiration and stent retriever (SR) has failed to show a benefit when compared to stent retriever alone [6]. However, despite its costs and technical challenges, it can be used both as a primary or alternative strategy. Although the choice of aspiration alone [7, 8] or with stent retriever [9] remains debatable, the access to the occlusion site remains an important factor for the recanalization success [10]. Furthermore, proximal flow arrest and reversal has been widely described as beneficial to successful recanalization despite chosen technique [11]. A large bore balloon guide catheter (BGC) that provides sufficient stability for tortuous vessels and enough navigability to enable a safe distal positioning seems to be associated with better technical outcomes [12]. One of the major limitations of current available BGC has been the incompatibility with large bore aspiration catheters (AC), forcing to compromise on either aspiration volume or flow arrest. The Walrus balloon-guided system catheter (Q’apel, Medical Inc., Fremont, CA) was proposed as an alternative to other BGC [12]. It is claimed to present variable stiffness to provide proximal support, high trackability to safely reach further distally and a large bore lumen to accommodate market-leading large bore aspiration catheters [13].

The purpose of this study is to provide the first European assessment of the Walrus BGC performance in a realistic, well-established in vitro simulation model [14] with different patient-specific cervical vascular anatomies.

## Material and methods

### Experimental setup

**Fig. 1:**
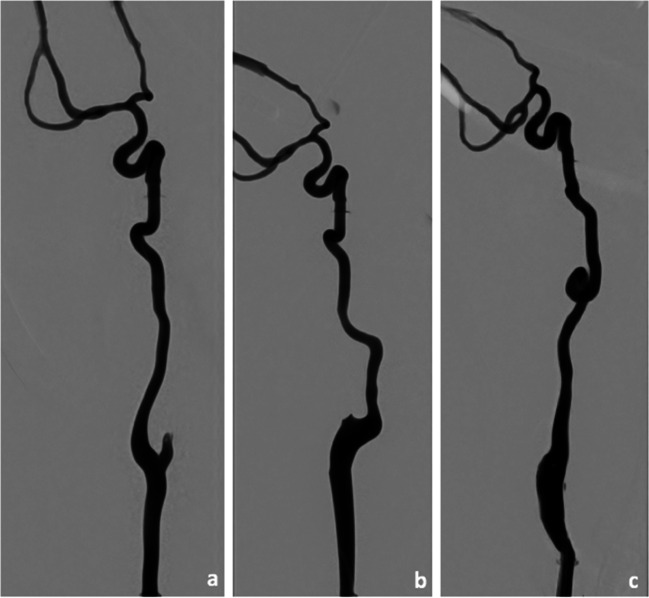
Custom-made patient-specific, 3D-fabricated anatomical variants of the ICA: straight (**a**), elongated (**b**), and looped (**c**)

A previously described modular neurovascular simulation model (HANNES) [15] with a commercially acquired silicone iliac-thoracoabdominal vasculature (United Biologics, Santa Ana, CA) and custom-made cervical and intracranial vessels were used. Patient-specific hollow vessel models based on anonymized patient data (ethic approval was waived by the local ethics committee) were produced by additive manufacturing using a commercially available 3D printer (Form 2, Formlabs, Somerville, MA, USA). The simulation system is integrated on a monoplane angiography system (AlluraClarity FD 20, Philips Healthcare, Best, The Netherlands). Three different anatomical variants (straight, elongated, and looped) of the cervical internal carotid artery (Fig. 1) were fabricated using a flexible resin (Flexible 80A, Formlabs, Somerville, MA) and attached to a skull base prototype. A physiological environment was simulated by an integrated fluid pump, equipped with a pulsatile valve and a heating system. The standard system configuration produced a flow rate of 0.4 L/min, a pulse rate of 70 bpm and a system temperature of 37 °C. Blood was substituted with a solution of water and small amount of commercially available soap for friction reduction. Synthetic clots [16] were placed in the M1-segment of the middle cerebral artery. Their position was confirmed by a single angiographic run using iodinated contrast medium. A custom-made distal emboli detection system was attached to the outflow channels.

### Devices

**Fig. 2:**
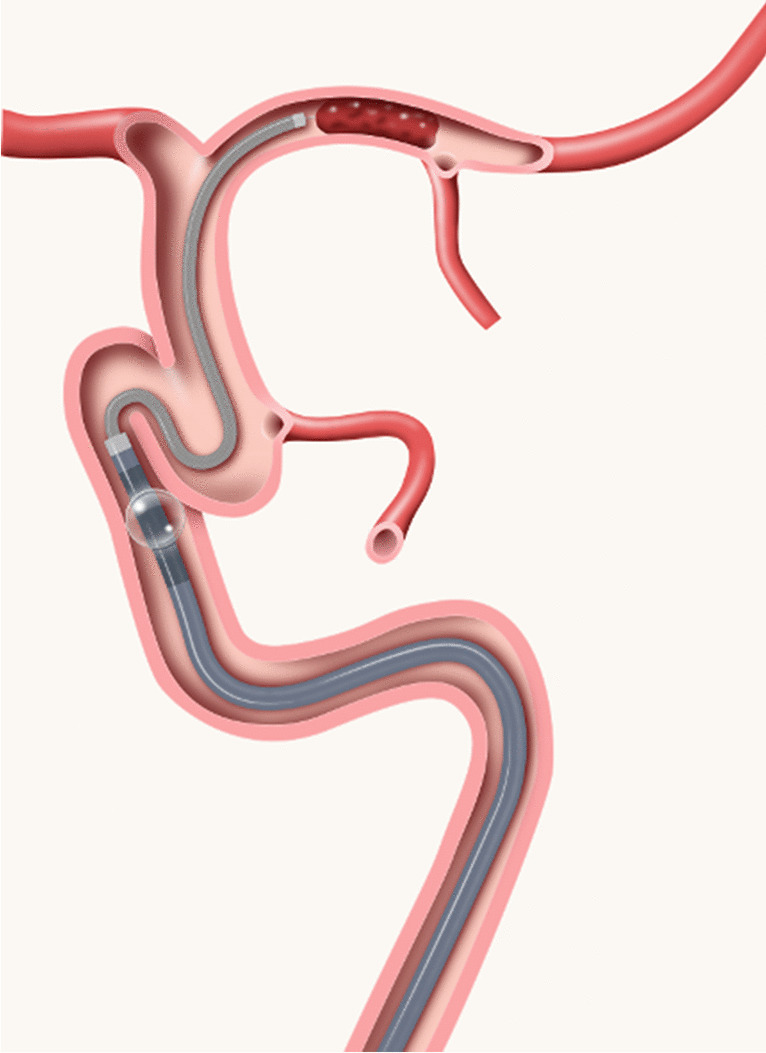
The figure illustrates a 0.087in ID, 95 cm long Walrus™ BGC with inflated balloon placed in the petrous segment of the ICA with a large bore AC (6F SofiaPlus™) placed in the MCA for contact aspiration. Courtesy of Q’apel Medical Inc.

The Walrus™ 087 BGC was compared with a commercially available large bore new generation FlowGate^2^™ (FG2) balloon guide catheter (Stryker, Kalamazoo, Michigan, USA). The FG2 used has an 8F/0.106in outer diameter (OD), an inner diameter of 0.084in (ID) and a length of 95 cm. The ID of the Walrus BGC is 0.087in, the OD 8F^+^/0.110in and equal length of 95 cm for comparison purposes. The diameters of the inflated balloons were 10 mm for the FG2 and 11.1 mm for the Walrus. Due to their compatibilities, different distal access catheters were used. With the FG2, a 5F Sofia™ (Microvention, Aliso Viejo, CA, USA) with an OD of 0.068in, ID 0.055in, and a working length of 125 cm was used. The Walrus™ could accommodate a 6F SofiaPlus™ with a proximal OD of 0.0825in, ID 0.070in, and length of 131 cm (Fig. 2). For aiding intracranial access, a Headway™ 0.021in ID microcatheter (Microvention, CA, USA) and a Traxcess™ 0.014in microwire (Microvention, CA, USA) were available when necessary.

### Procedure details

The balloon guide catheters were advanced through a short 9F femoral sheath in the right femoral artery and advanced to the common carotid artery over a 5F SIM2 125 cm catheter (Cordis, Miami Lakes, FL, USA) for navigability. Seven interventional neuroradiologists with an average experience of 9.9 years in mechanical thrombectomy (BE 15, CB 13, FW 12, TI 10, MB 10, FF 7, HG 2) performed one recanalization attempt per anatomical variant by direct aspiration with the two different catheter combinations: Walrus/Sofia6F and FG2/Sofia5F. Operators were instructed to place the BGCs as distal as comfortably possible. The segmental level of the ICA [17] reached by the BGC, access to occlusion site, time to delivery, induced anatomical change and catheter kick-back were registered.

### Evaluation and statistical analysis

The participants responded to a questionnaire composed of 8 questions and an ordinal classification scale with a 5-point system (1 to 5). Responses were collected with the survey online platform Forms.App (Tallin, Estonia). Further data collection was performed on Microsoft Excel for Mac (Redmond, WA, USA). All statistical analyses were performed with SPSS (IBM, Chicago, IL, USA). Continuous variables were analyzed using Student’s *t*-test for two samples; comparison across operators and ICA models was performed with ANOVA. Nonparametric variables were analyzed using chi-square independence test, the Mann–Whitney *U*-test for two independent variables, and Kruskal–Wallis test for more than two independent groups.

## Results

### Mechanical thrombectomy simulation

**Fig. 3:**
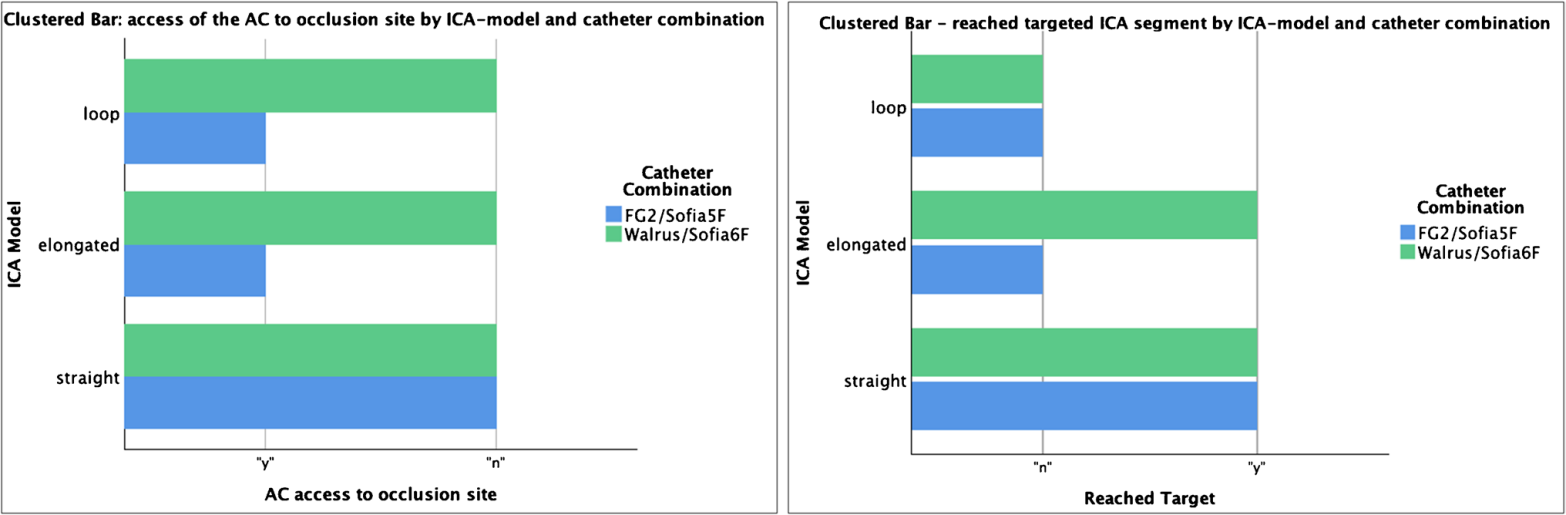
Clustered bar graphs representing significant better access of the Walrus BGC/6F SofiaPlus to occlusion site despite vessel anatomies (left) and to the petrous segment of ICA (right). In the looped vessel variant, a navigation of the BGC to the distal segments of the cervical segment was not possible

A total of 41 thrombectomies were performed by the operators with three levels of access complexity by means of a straight ICA (*N* = 15), elongated (*N* = 16), and looped (*N* = 10) ICA anatomy. The target for placement of the BGC was considered the petrous segment of the ICA (levels C2 or above according to Bouthillier [17]). The Walrus™ (45%, 10/22) was significant more likely to reach distally to target level (*p* = 0.04) than the FG2 (15.8%, 3/19), reaching in 7/10 (70%) cases the C2 level and in 3/10 (30%) the C3 level vs. 2/3 (67%) the C2 and 1/3 (33%) the C3 level for the FG2. In 2 cases, the same operator did not attempt to reach target level due to his current practice. The occlusion site could be accessed by the Walrus/Sofia6F in 19/22 (86%) cases, whereas the FG2/Sofia5F only reached the occlusion in 5/19 (26%) cases with a significance *p* < 0.001 (Fig. 3). The time required to access the site of occlusion did not differ significantly across ICA models or catheter combinations. However, this differed significantly among operators (*F* = 3.35; *p* = 0.011). Anatomical change caused by catheter manipulation or catheter kick-off upon distal access of the AC did not differ significantly among catheter combinations or vessel models. The degree of anatomical complexity did not play a significant role in the access to the occlusion site. The delivery of the BGC to the target segment (Fig. 3) was significantly (*p* < 0.01) higher in the straight ICA (66.7%, 10/15) in comparison to the elongated (18.8%, 3/16) or looped ICA (0%, 0/10). In one case, only distal emboli were detected after balloon rupture (FG2).

### Qualitative evaluation

**Fig. 4:**
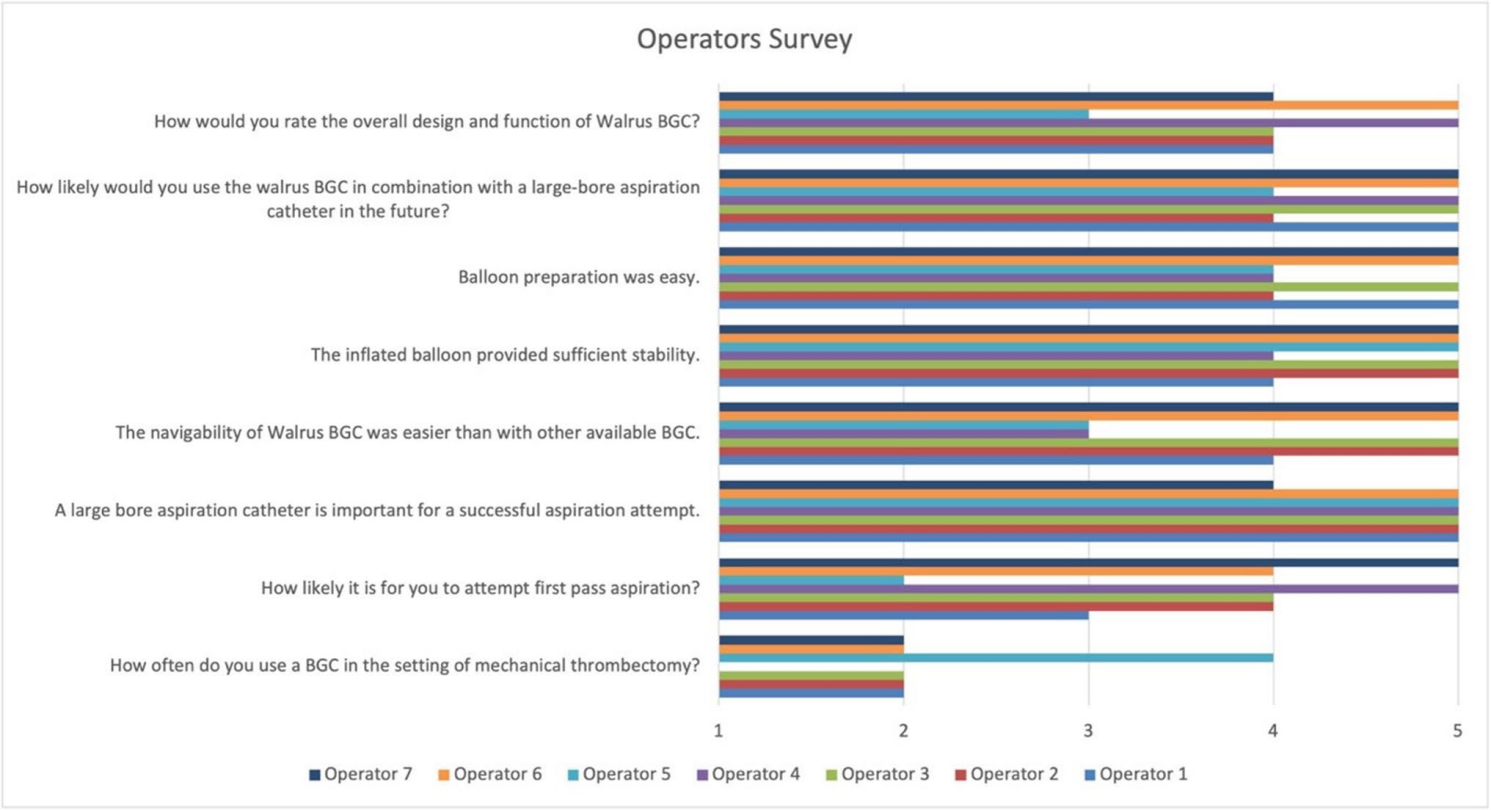
Results of the questionnaire with the operators’ qualitative assessment after the in vitro experiments. Each operator provided ordinal scale ratings ranging from 1 to 5 for each question (1–strongly disagree/never/very unlikely/very poor; 2–disagree/almost never/unlikely/poor; 3–somewhat agree/sometimes/neutral/acceptable; 4–agree/often/likely/good; 5–strongly agree/always/very likely/very good)

Most operators stated that they almost never (71.4%, 5/7) or never (14.3%, 1/7) use a BGC in the setting of MT. 3/7 operators (42.9%) will likely or very likely (2/7, 28.6%) attempt direct aspiration (Fig. 4). A large bore aspiration catheter was unanimously considered important (85.7%, 6/7 strongly agree and 14.3%, 1/7 agree). The navigability with the Walrus™ was considered easier than that of other BGCs (57.1%, 4/7 strongly agree; 28.6%, 2/7 somewhat agree and 14.3%, 1/7 agree) and the balloon sufficiently stable upon inflation (71.4%, 5/7 strongly agree and 28.6%, 2/7 agree). Balloon preparation was considered easy (57.1%, 4/7 strongly agree and 42.9%, 3/7 agree). All operators would like to use the Walrus™ BGC in the future (71.4%, 5/7 strongly agree and 28.6%, 2/7 agree). The overall design was rated as very good (28.6%, 2/7), good (57.1%, 4/7), or acceptable (14.3%, 1/7).

## Discussion

This study describes the first experience with a novel, to the current day in the European Union not yet commercially available BGC, in a realistic stroke simulation model. The Walrus™ BGC has been described as a promising alternative to other widely available BGCs. In previous studies, it showed a safe and excellent distal navigability, not compromised by a large bore, capable of accommodating the largest AC available [12]. Our in vitro observations align with the previous studies [13]. The Walrus™ BGC was able to navigate further distally and the combination with a 131 cm 6F SofiaPlus™ AC, was significantly more likely to access the occlusion site for direct aspiration. To this day, a 5F Sofia™ AC with equivalent length is not yet available, which represents a considerable limitation in the access of occlusion sites, especially in elongated vasculatures. However, access to the occlusion site did not differ among different anatomies, based on which, we could postulate that despite vascular tortuosity, a better BGC navigability and higher AC length, may be decisive to successfully reach the occlusion. Different access times among operators reflect the different degrees of technical proficiency and experience with the in vitro model. Catheter kick-back and degree of anatomical change caused by the catheters were unlikely to be true to reality, due to the comparatively rigid vessel models.

BGCs provide a flow arrest and reverse flow during MT, which reduces the risk of clot fragmentation and distal embolization [13]. The use of BGC has been associated with shorter procedures, higher rates of successful revascularization and higher rate of one-pass thrombectomy [18, 19]. Baek et al. described the use of BGC as an independent factor for both recanalization success and clinical outcome, despite of the used primary endovascular modality [20]. Furthermore, TICI 3 recanalization has been reported nearly 20% more likely in MT using a BGC than in those without BGC [21]. Nevertheless, BGCs present some well-known limitations. In spite of their large 8F outer diameter, they are incompatible with new large bore aspiration catheters [22], forcing a compromise in aspiration volume. Yet, greater aspiration volumes enabled by ACs with larger diameters seem to be associated with better reperfusion rates [23]. Concerns over associated costs, navigability, tractability, and risk of vessel dissection during navigation have limited the widespread establishment of BGC as a technical standard in MT and consequently, the use of BGC has been reported only in 45–50% of MT across thrombectomy registries [19, 24]. BGCs are less likely to be used in patients with tortuous vessel anatomy [21] due their stiffness and limited navigability. However, a retrospective study reported better recanalization rates of about 30% in patients with a distal vs. proximal BGC placement in cervical ICA [25]. The appropriate catheter length, proximal stability, and sufficient distal flexibility are necessary for safer distal navigation. The specially designed monolithic wall of the Walrus™ BGC is claimed to offer a better navigability and adaption to a curved anatomy with improved resistance to kink [12].

This study has several limitations. It is a simulation-based study performed in a flow model under stable physiological conditions, which may differ from those found in patients during the acute setting. The vessel models are less malleable in comparison to human vessels, which most likely influence the navigation and positioning of the catheters. Although flow arrest can be achieved, due to technical features of the simulation model, flow reversal cannot. Available clots presented variable compositions and their placement in the flow model was slightly variable, causing unpredictable difficulty in occlusion passage and thus, the results of thrombectomy were not taken into consideration. High clot rigidity may have influenced the reduced number of distal emboli detected. Not all experiments were performed in equal numbers due to limited operator availability. Operators were not blinded to the technique and material used. The operators were instructed to navigate as distally as possible, which invariably affected the results. The relatively small number of experiments represented a further limitation of this study.

## Conclusions

In an in vitro experimental setting, the Walrus™ BGC provided excellent distal navigability, good access to the occlusion site and did not implicate a compromise on aspiration volume. Its use may contribute to the wider adoption of balloon guide catheters in the setting of mechanical thrombectomy. Further prospective trials to determine how this may affect recanalization rates are urged.

## Data Availability

The data that support the findings of this study are available from the corresponding author (HG) upon reasonable request.
